# Tracking human multiple myeloma xenografts in NOD-Rag-1/IL-2 receptor gamma chain-null mice with the novel biomarker AKAP-4

**DOI:** 10.1186/1471-2407-11-394

**Published:** 2011-09-16

**Authors:** Leonardo Mirandola, Yuefei Yu, Marjorie R Jenkins, Raffaella Chiaramonte, Everardo Cobos, Constance M John, Maurizio Chiriva-Internati

**Affiliations:** 1 Division of Hematology & Oncology, Texas Tech University Health Sciences Center and Southwest Cancer Treatment and Research Center, Lubbock, TX, USA; 2 The Laura W. Bush Institute for Women's Health and Center for Women's Health and Gender-Based Medicine, Texas Tech University Health Sciences Center, Amarillo, TX, USA; 3 Departments of Internal Medicine and Obstetrics & Gynecology, Texas Tech University Health Sciences Center, Amarillo, TX, USA; 4 Department of Medicine, Surgery and Dentistry, Università degli Studi di Milano, Milano, Italy; 5 MandalMed, Inc., San Francisco, CA, USA

## Abstract

**Background:**

Multiple myeloma (MM) is a fatal malignancy ranking second in prevalence among hematological tumors. Continuous efforts are being made to develop innovative and more effective treatments. The preclinical evaluation of new therapies relies on the use of murine models of the disease.

**Methods:**

Here we describe a new MM animal model in NOD-Rag1null IL2rgnull (NRG) mice that supports the engraftment of cell lines and primary MM cells that can be tracked with the tumor antigen, AKAP-4.

**Results:**

Human MM cell lines, U266 and H929, and primary MM cells were successfully engrafted in NRG mice after intravenous administration, and were found in the bone marrow, blood and spleen of tumor-challenged animals. The AKAP-4 expression pattern was similar to that of known MM markers, such as paraproteins, CD38 and CD45.

**Conclusions:**

We developed for the first time a murine model allowing for the growth of both MM cell lines and primary cells in multifocal sites, thus mimicking the disease seen in patients. Additionally, we validated the use of AKAP-4 antigen to track tumor growth *in vivo *and to specifically identify MM cells in mouse tissues. We expect that our model will significantly improve the pre-clinical evaluation of new anti-myeloma therapies.

## Background

According to the American Cancer Society, more than 20,000 patients were diagnosed with multiple myeloma (MM) in the US in 2010. Among hematologic malignancies, MM ranks second in prevalence and has the shortest 5-year survival rate [1]. Multiple myeloma (MM) is an age-related cancer caused by the accumulation of antibody-producing malignant plasma cells and leads to progressive osteolysis, defective hematopoiesis and renal failure [2]. Recent progresses in understanding the molecular bases of MM have lead to the use of innovative drugs, such as bortezomib, thalidomide and lenalidomide [3]. Unfortunately, although these therapies afforded a significant improvement in the disease course, MM remains invariably fatal because of the high rate of multidrug-resistant relapse [4]. On these bases, constant efforts are dedicated to the evaluation of more effective treatment strategies [5-7].

Similarly to other malignancies [8], virtually any innovative treatment for MM requires a pre-clinical assessment, which largely relies on the use of animal models to evaluate the anti-tumor potential and possible toxicities [9-12]. To this goal, sub-lethally irradiated immunodeficient NOD/SCID mice have been extensively used since they allow for human MM cell line xenografting after intravenous injection [13-23]. More recently, it has been shown that NOD/SCID mice carrying nonfunctional IL-2 receptor gamma chain (NOD/SCID/γc^null^, NOG) are more permissive recipients than NOD/SCID and can be easily xenografted with human MM cell lines to produce a disease similar to that seen in patients, including multiple metastatic sites and bone lesions [24,25]. A further modification of the NOD strain, carrying double genetic disruptions of the Rag1 and the IL-2 receptor gamma chain genes, namely NOD-Rag1^null ^IL2rg^null ^(NRG), has been reported to tolerate higher levels of radiation compared with NOD/SCID and NOG strains and to allow for efficient engraftment of human hematopoietic stem cells [26].

The development of successful animal models for MM also relies on the choice of the biomarkers used to track the disease course and to identify tumor cells in mouse tissues [27-32]. The A-kinase anchor protein 4 (AKAP-4) [33] is a scaffolding protein that participates in the intracellular signaling of protein kinase-A [34]. AKAP-4 is a cancer/testis antigen (CTA), a class of tumor associated antigens characterized by high expression in germ cells and cancer, strong immunogenicity and very low expression or absence in normal tissues [35,36]. We have previously shown that AKAP-4 is abnormally expressed at the mRNA and protein levels in MM cell lines and patients' MM primary cells, but absent in normal tissues, and therefore it is a potential novel biomarker for MM [37].

In this study, we used for the first time the NRG strain to establish an innovative model of MM, allowing for the growth and the spread of MM cell lines and primary patients' cells as well. Additionally, we provide evidence that the CTA AKAP-4 is a reliable and specific biomarker that can be used to track the growth of MM cell lines and primary cells *in vivo*.

## Results

### Detection of tumor growth in vivo by ELISA

**Figure 1:**
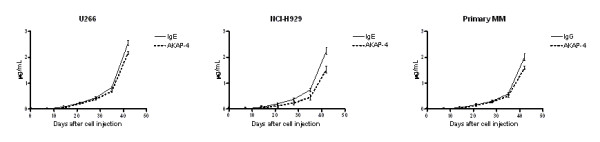
**Measurement of circulating paraproteins and AKAP-4 levels**. Mice were bled once a week as described in the Methods section. The assay was run in triplicate for each time point. Graphs display mean protein levels obtained from each tumor group and error bars indicate SEM. No statistically significant difference was evidenced between IgE or IgG and AKAP-4 levels at any of the analyzed time points as evaluated by two-way ANOVA (Bonferroni's post-test p > 0.05 for all comparisons).

Indirect ELISA was used to determine the concentration of human paraproteins (IgE and IgG) and AKAP-4 in the sera of tumor-bearing mice (Figure 1). Anti-human IgE antibodies were used to monitor the growth of U266 and H929 [38], since they are IgE-producing cell lines. For MM primary cells, IgG was used as a paraprotein marker [39]. Figure 1 shows that paraprotein and AKAP-4 levels became evident starting 21 days after injection, and that a progressive increase was detectable over time. Although AKAP-4 levels were on average 20% lower than IgE and IgG, no significant difference between AKAP-4 and paraprotein mean levels was detected at any time analyzed point (two-way ANOVA and Bonferroni's post-test p > 0.05).

### Flow-cytometry identification of MM cells from mouse tissues

**Figure 2:**
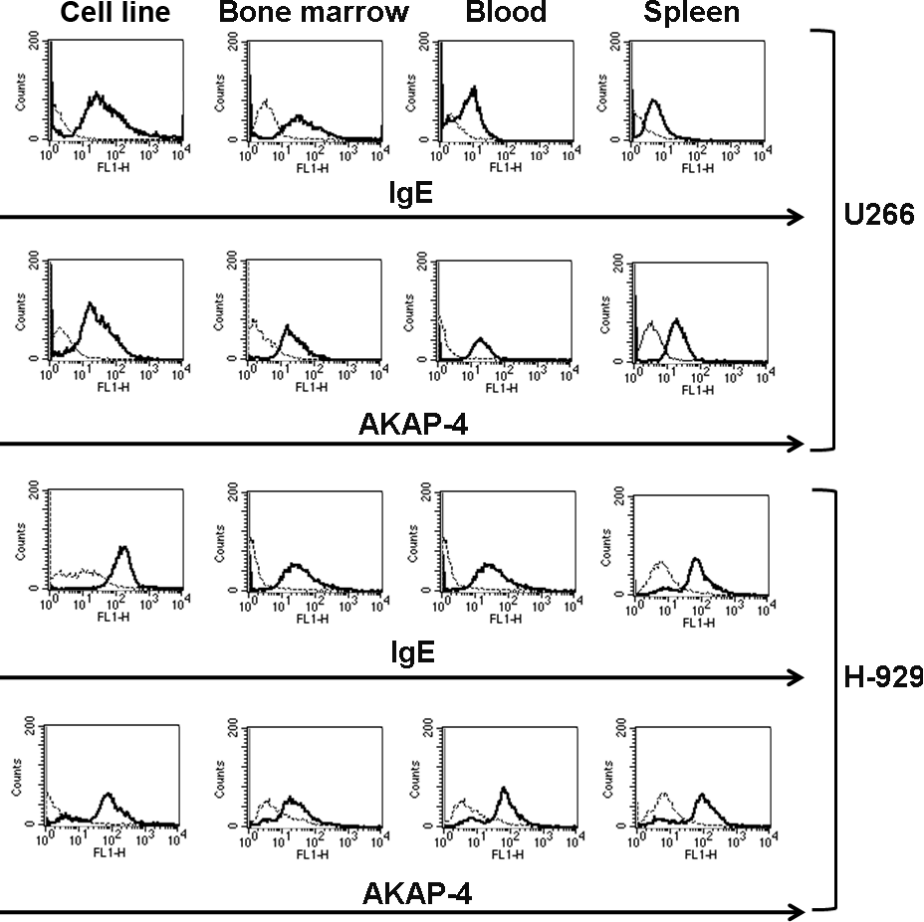
**Detection of MM cell lines in tumor-bearing mice**. U266 and H929 cell lines, or tissues derived from U266 and H929- injected mice analyzed by flow-cytometry. Histograms show the fluorescence intensity measured with the indicated specific antibody (bold lines) or with the corresponding isotypic control (dotted lines). Graphs are representative of comparable results obtained from 5 U266- and 5 H929-challenged mice.

**Figure 3:**
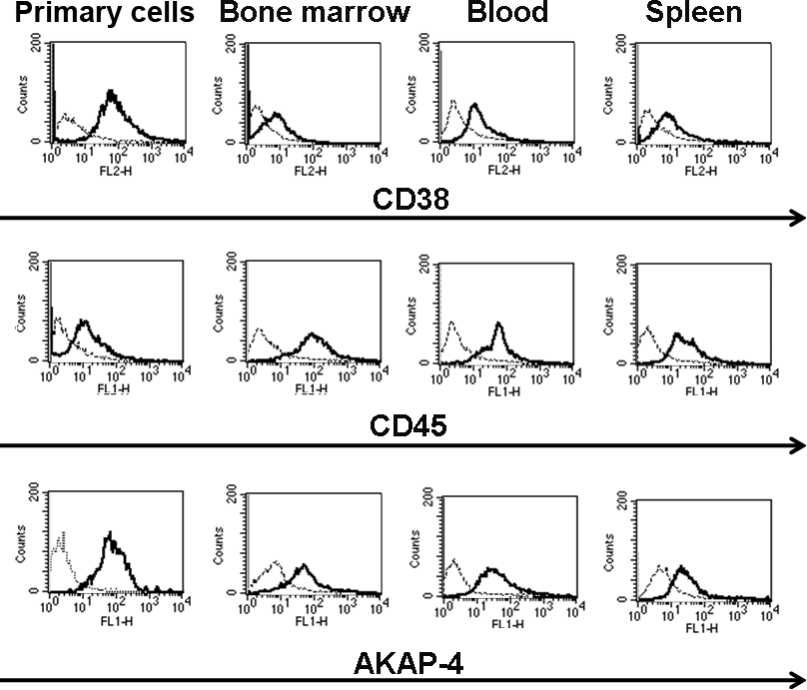
**Detection of primary MM cells in tumor-bearing mice**. Ficoll-hypaque isolated cells from primary bone aspirate, or cells derived from primary MM-injected mice were analyzed by flow-cytometry. Histograms show the fluorescence intensity measured with the indicated specific antibody (bold lines) or with the corresponding isotypic control (dotted lines). Histograms are representative of comparable results obtained from 5 primary MM-challenged mice.

**Figure 4:**
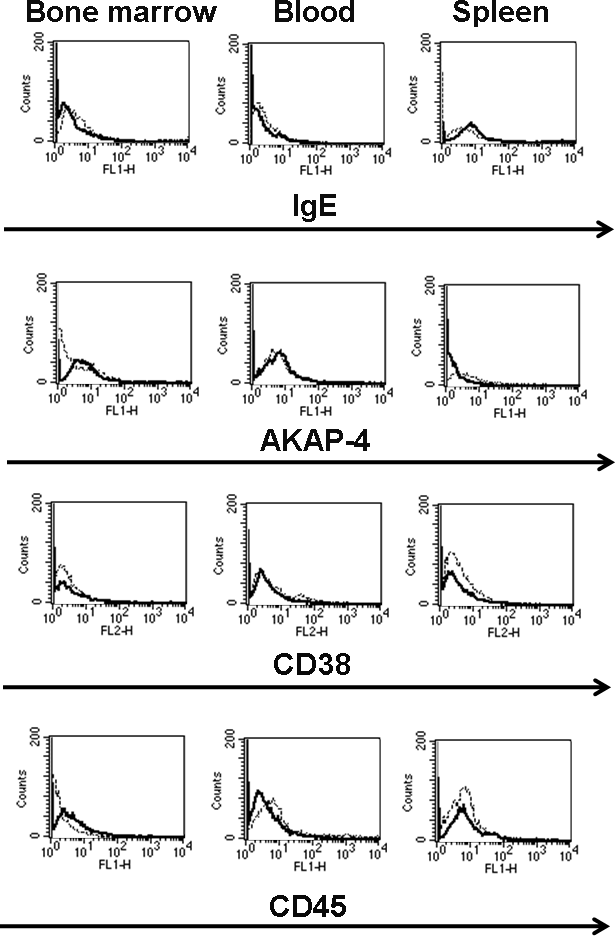
**Flow-cytometry evaluation of tumor-free mice**. Six weeks after irradiation, healthy control mice were euthanized and processed in parallel with tumor-bearing mice. Histograms show the fluorescence intensity measured with the indicated specific antibody (bold lines) or with the corresponding isotypic control (dotted lines). Histograms are representative of comparable results obtained from 5 tumor-free mice.

Six weeks after initiation of tumor challenging, tumor-bearing and healthy mice were euthanized, and tissues were processed as described in the Methods section. Flow cytometry analysis was performed to detect the presence of MM cell lines or primary cells in the bone marrow, blood and spleen (Figures 2, 3 and 4; Tables 1, 2, 3). Exponentially growing U266 and H929 cell lines or primary cells from bone marrow aspirate were used as positive controls (Figures 2 and 3; Tables 1, 2). IgE was used as a marker for U266 and H929 [38], while primary MM cells were identified by CD38 and CD54 [39]. AKAP-4 was expressed by MM cell lines (Figure 2; Tables 1, 2) and primary MM cells (Figure 3; Table 3); therefore it was tested for the detection of both cell types. Results showed that IgE^+ ^U266 and H929 were present in mouse bone marrow, blood and spleen (Figure 2; Tables 1, 2). Similarly, primary CD38^+ ^and CD54^+ ^primary MM cells were detected in bone marrow, blood and spleen (Figure 3; Table 3). The expression pattern of AKAP-4 was comparable to that of IgE, CD38 and CD54 (Figures 2 and 3; Tables 1, 2, 3). The specificity of the assay was confirmed by the failure to detect positive cells in tumor-free mice (Figure 4).

**Table 1:** Mean fluorescence intensity for U266 cells

Marker	Cell line	Bone marrow	Blood	Spleen
**IgE**	56.3^a ^(5.4)^b^	48.7 (3.8)	25.4 (3.2)	19.7 (4.5)

**AKAP-4**	54.9 (3.3)	47.8 (7.6)	52.1 (6.4)	38.7 (4.5)

Mean fluorescent intensities (a) and SEM (b) were calculated by flow cytometry from cells and tissues of 5 U266-challenged mice.

**Table 2:** Mean fluorescence intensity for H929 cells

Marker	Cell line	Bone marrow	Blood	Spleen
**IgE**	135.7^a ^(12.8)^b^	49.6 (5.3)	46.2 (7.3)	128.9 (14.3)

**AKAP-4**	131.5 (7.6)	45.2 (8.1)	128.7 (5.7)	131.2 (6.4)

Mean fluorescent intensities (a) and SEM (b) were calculated by flow cytometry from cells and tissues of 5 H929-challenged mice.

**Table 3:** Mean fluorescence intensity for primary MM cells

Marker	Cell line	Bone marrow	Blood	Spleen
**CD38**	98.9^a ^(11.2)^b^	22.7 (6.7)	27.8 (8.4)	25.4 (4.7)

**CD45**	26.7 (3.1)	97.6 (7.6)	87.2 (4.1)	38.7 (2.1)

**AKAP-4**	98.6 (8.7)	88.4 (7.6)	33.2 (2.4)	47.6 (5.3)

Mean fluorescent intensities (a) and SEM (b) were calculated by flow cytometry from cells and tissues of 5 primary MM-challenged mice.

### Analysis of AKAP-4 expression at the mRNA and protein levels in MM xenografts

**Figure 5:**
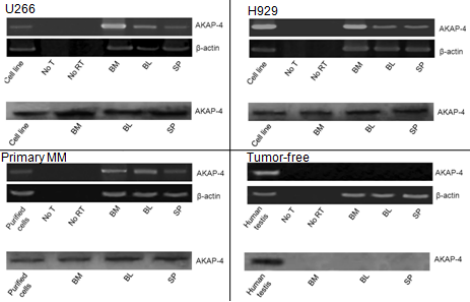
**RT-PCR/Western blot analyses of AKAP-4 mRNA/protein expressions and**. RT-PCR analysis of mouse tissues was performed to evaluate AKAP-4 expression. AKAP-4 was detected in bone marrow (BM), blood (BL) and spleen (SP) of mice injected with MM cell lines (U266, H929) and primary tumor cells, but not in tumor-free mice. Positive controls were RNA isolated from exponentially *in vitro *growing cell lines, or from primary cells after ficoll purification. For the evaluation of healthy mice, RNA isolated from human testis was used as a positive control. Analysis of β-actin transcript served to confirm RNA integrity. Negative controls were PCR performed without template (No T), or with RNA not subjected to the reverse transcription step (No RT). Proteins extracted from MM cells and mouse tissues were analyzed for the presence of AKAP-4 protein by Western blot. AKAP-4 protein was detected in MM cell lines (U266 and H929), primary MM cells, and in the bone marrow, blood and spleen of tumor-challenged mice, but not in healthy controls (tumor-free). Total protein extracts from human testis were used as a positive control for the analysis of tumor-free mice. The pictures are representative of comparable results independently obtained from 5 mice in each group.

RT-PCR was performed to evaluate AKAP-4 mRNA expression in tumor-challenged or tumor-free mice. Results (Figure 5) show that the AKAP-4 transcript was present in MM cell lines, primary MM cells, bone marrow, peripheral blood and spleens of tumor-bearing mice, but undetectable in tumor-free mice (healthy controls). Specificity of results was also confirmed by PCR reactions carried out without cDNA template or without retrotranscribed RNA.

AKAP-4 protein was detected by Western blot analysis (Figure 5) in MM cell lines, primary MM cells, bone marrow, peripheral blood and spleens of tumor-bearing mice, but not in tumor-free mice (healthy controls).

## Discussion

This study was aimed to establish and characterize a new murine model of disseminated MM, allowing for the engraftment of human MM cell lines and primary tumor cells derived from MM patients. To this goal, we used the NOD-Rag1^null ^IL2rg^null ^(NRG) murine strain, intravenously injected with MM cell lines or with primary MM cells. The lacking of a functional IL-2 receptor makes IL2rg^null ^mice better xenograft recipients then NOD/SCID animals, because of the absence of NK cells [38]. In addition, compared with NOD/SCID or NOD/SCID/γc^null ^(NOG) strains, NRG mice tolerate significantly higher levels of radiation. Differently from SCID mice, the NRG strain carries a functional Prkdc gene, which is essential for the repair of DNA damage induced by radiation in many tissues [26].

ELISA for serum MM paraproteins showed that xenografted animals supported the growth of both MM cell lines and primary tumor cells. Importantly, AKAP-4 was detectable in the sera of tumor-challenged mice and its levels increased over time, similarly to those of IgE and IgG. This indicates that AKAP-4 is a suitable biomarker for tracking MM progression in murine xenografts. Different techniques have been described to monitor the MM burden in animal models, such as fluorescent tagging of tumor cells [40-45] or measurement of MM-derived paraptrotein in the serum [29-32]. In the clinic, better methods for staging and monitoring the aggressiveness of MM, especially in assessing relapse, are thought to be critical to improve patients' outcomes and develop personalized therapies [46]. A number of methods are under investigation, including mass spectrometry for the quantification of serum immunoglobulins [46], and immunohistochemistry for the expression of FGFR3 and cyclin D1 (reported in 15%, and 50% of patients with MM, respectively) [46,47]. In this context, the identification of novel tumor antigens in the sera could be instrumental for a more sensitive detection of disease progression [36]. Here we showed for the first time the use of AKAP-4 as a novel serum biomarker in MM animal models. Further investigations are warranted to evaluate AKAP-4 serum levels in MM patients and the correlation with treatment outcome.

Flow-cytometry analysis confirmed the presence of MM cell lines and primary patient's cells in the bone marrow, blood and spleen of tumor challenged mice, indicating that intravenously injected tumor cells were able to systemically disseminate *in vivo*. The specificity of this finding was confirmed by the failure to detect paraprotein- or AKAP-4- positive cells in tumor-free mice. Additionally, we showed that AKAP-4 was expressed in the same tissues at the transcriptional and protein levels in tumor-bearing animals, but absent in healthy controls. Collectively, these results indicate that our model is suitable for the growth and systemic dissemination of human MM cell lines and primary tumors. Currently available murine models for MM include immunocompetent mice, such as the 5TMM series [48,49] and genetic models of MM [50-52], or immunocompromised mice, namely NOD/SCID [13-23], SCID-hu [53-56], and NOG [24,25,38,57]. The 5TMM and the genetic models of MM have the advantage of affording pre-clinical studies in immunocompetent hosts, where possible effects of the therapy on the interaction between tumor cells and the immune system can be evaluated. However, molecular and biological differences exist between murine and human MM cells [11]. Additionally, the number of available murine genetic models of MM and of 5TMM cell lines is extremely restricted and do not represent the heterogeneity of the human disease [50-52,58,59]. Therefore, it is evident that pre-clinical studies on MM cells of human origin are essential [9], but they are only feasible by using immunodeficient murine xenografts. Among these, subcutaneous inoculation of human MM cells has been extensively described [13,17,45,60-62]. This model affords the possibility to directly assess changing in tumor growth induced by therapies. Yet, tumor cells growing subcutaneously do not interact with the bone marrow microenvironment, which largely accounts for MM drug resistance [61,63]. Because we described the presence of tumor cells in the bone marrow of tumor-challenge mice, we propose that our model is suitable to evaluate the protective role played by the bone niche against anti-tumor therapies. Some concerns have been raised about the possibility that the interactions between MM cells and the bone stroma may be partially species-specific [11,64]. To address this potential difficulty, the SCID-hu model was developed, in which MM cells are located in subcutaneously implanted human bone chips [53-56]. Although SCID-hu mice allow for the growth of MM cells in a bone niche of human origin [53-56], they do not reproduce the pattern of dissemination and multifocal spread seen in MM patients. Here we described for the first time a murine model supporting the engraftment human MM cells, and allowing for the development of a disease involving multiple sites, similar to that observed in patients [65-73]. This is an important advantage, because the effect of bone resorption in multiple osteolytic lesions is a critical factor for the survival of MM patients [6,7]. Pre-clinical studies basing on immunocompromised xenograft models have previously described metastatic spread involving multiple bones, yet they have been limited to human MM cell lines [40,41,43,44,74-76]. In this study, we show that our model supports the metastatic growth of primary human MM cells. MM cell lines do not adequately represent the heterogeneity of the human disease because they are established from late stage disease and frequently present mutations not seen in patients [9]. Therefore, the possibility to study not only cell lines, but also primary MM cells in murine models is relevant.

## Conclusions

We presented here the proof-of-principle for the use of NRG mice as a new model supporting the metastatic growth of human MM cell lines and primary cells. Additionally, we propose the use of AKAP4 as a universal biomarker to track tumor cells *in vivo*. We foresee that our results will significantly contribute to the improvement of the pre-clinical evaluation of new anti-myeloma therapies. Because our model sustains the growth of primary MM cells, further investigations are warranted to study the suitability of this system to assess the efficacy of personalized therapies directly on patient's cells.

## Methods

### Animals

Six-week-old female NOD.Cg-Rag1^tm1Mom ^IL2rg^tm1Wjl^/SzJ (NRG) mice were obtained from the Jackson Laboratory (Bar Harbor, ME, U.S.A.). All mice were maintained in filtered-air laminar-flow cabinets under specific pathogen-free conditions. Treatment and care of the animals were in accordance with the Institutional Guidelines and the Animal Welfare Assurance Act. The mice were checked daily and euthanized 6 weeks after tumor challenge or if they showed signs of excessive discomfort (hind leg paralysis, inability to move, eat or drink).

### Human MM cell lines

The human MM cell lines U266 and H929 were purchased from the American Type Culture Collection (Manassas, VA, U.S.A.), and cultured in RPMI-1640 medium, supplemented with 10% V/V fetal bovine serum (FBS) and penicillin/streptomycin mix (10 mg/mL each) in 95% air and 5% CO_2 _at 37°C. Prior to injection, cells were washed once in PBS and then resuspended at 10^8 ^cells/mL in pre-warmed PBS prior to injection.

### Primary MM cells

Human material was obtained under informed consent and with the approval from the local ethics committee. Bone marrow aspirate was obtained from a MM patient at diagnosis (Durie-Salmon stage III) from the hip bone. Light density cells were separated by ficoll hypaque centrifugation (Histopaque; Pharmacia, Uppsala, Sweden) [39], washed twice in PBS, counted and adjusted at the final concentration of 10^8 ^cells/mL in pre-warmed PBS prior to injection.

### Xenografts

20 mice were sub-lethally irradiated with a total dose of 550 cGy at 139 cGy/min rate [26]. After 6 hours, mice were assigned to the following groups (5 mice/group): group 1 received U266 cells, group 2 received H929, group 3 was given primary MM cells, while group 4 was left tumor-free and served as a negative control. Each mouse received 10^7 ^cells by a single intravenous injection in the lateral tail vein (100 μL/mouse).

### ELISA for the measurement of serum paraprotein and AKAP-4 concentration

Blood (50 μL) was collected weekly from each mouse. Serum was prepared by centrifugation in the absence of anticoagulants and stored at -20°C until use. An enzyme-linked immunosorbent assay (ELISA) was performed on mouse sera for the determination of human paraprotein (IgE and IgG) [39] or AKAP-4 concentration. Antibodies were purchased from BD Biosciences (San Diego, CA, U.S.A.). 96-well polystyrene plates were coated with serum (50 μL/well diluted 1:10 in carbonate coating buffer), and incubated overnight at 4°C. Plates were washed three times in PBS containing 0.05% (V/V) Tween-20 (PBS/Tween) and then incubated in 1% bovine serum albumin (BSA) in PBS for 1 h at RT to block unspecific sites. After washing three times with PBS/Tween, plates were incubated at 37°C with 50 μL/well of primary anti-human IgE, IgG, or AKAP-4 antibodies (5 μg/mL in PBS) for 1 h. After washing twice with PBS/Tween, HRP-linked secondary antibody (1:4,000 dilution in PBS, 50 μL/well, Santa Cruz Biotechnology, CA, U.S.A.) was added and allowed to bind for 60 minutes at RT. After washing trice with PBS/Tween, 100 μL/well of TMP substrate (Abcam, Carpinteria, CA, U.S.A.) was added. The reaction was stopped 15 minutes later by adding 50 μL/well H_2_SO_4 _solution. Optical density (OD) was measured with a Victor^2 ^plate reader (PerkinElmer, Waltham, MA) at 450 nm. All samples were analyzed in triplicates. Quantification of the target antigens was made by interpolation of the mean OD for each sample using a standard curve obtained by 13 serial 3-fold dilutions (from 2,400 to 1.5 × 10^-3 ^ng/mL) of purified human paraproteins (GenWay Biotech, Inc., San Diego, CA, U.S.A.), or human AKAP-4 (Abnova, CA, U.S.A.).

### Preparation of tissues

Femurs, hips, sternums, and spleens were mechanically disrupted in serum-free RPMI-1640 medium. Minced organs were placed into 250 mL flasks containing 3 mL of enzyme solution (0.14% collagenase type I; Sigma Aldrich, MO, U.S.A.) and 0.01% DNase (2000 kU/mg; Sigma Aldrich) in RPMI-1640 and incubated on a magnetic stirring apparatus at 37°C for 30 min. Then, cells were washed in PBS and filtered through a nylon mesh with 150 μm pores to generate single-cell suspensions. Blood (500 μL) was taken by retro-orbital venipuncture immediately after the euthanasia procedure and placed in a heparin-coated tube. Cells were harvested by centrifugation and washed twice in PBS before analyses.

### Flow-cytometry

The expression of human IgE, CD38, CD45 and AKAP-4 was analyzed by flow-cytometry 6 weeks after tumor injection as previously described [77]. Specifically, IgE was used to identify U266 and H929 cells [38], while CD38 and CD45 were used as markers for human MM primary cells [39]. AKAP-4 was analyzed in both cell lines and primary cells [37]. U266, H929, primary MM cells and cells obtained from mouse bones, spleen and blood were fixed with 2% W/V buffered PFA (Sigma-Aldrich, MO, U.S.A) in PBS for 5 minutes at RT. After washing with PBS, cells were permeabilized with 0.3% saponin (Sigma-Aldrich, MO, U.S.A.) in PBS for 5 minutes at RT. After washing twice with PBS, cells were incubated on ice with monoclonal antibodies raised against human IgE, CD38, CD45 or AKAP-4 (Santa Cruz Biotechnology, CA, U.S.A.) or isotypic controls for 1 hour. After washing twice with PBS, cells were incubated with FITC-conjugated secondary antibodies (for IgE, AKAP-4 and CD45), or PE-conjugated secondary antibody (for CD38) (BD Biosciences, NJ, U.S.A.) for 1 h on ice. Analysis was performed using a BD FACScan (BD Biosciences, NJ, U.S.A), after 3 final washing steps with PBS.

### RT-PCR and immunoblot

Total RNA was extracted from bone, spleen, blood, or from MM cell lines and primary MM cells by Trizol-reagent (Sigma, St Louis, MO, U.S.A.). Purified total RNA was treated with 5 μg RNase-free DNase I (Promega, Madison, WI, U.S.A.) at 37°C for 2 h. mRNA was then isolated using Oligotex mRNA Mini Kit (QIAGEN, Valencia, CA, U.S.A.). First-strand cDNA synthesis was performed using oligo (dT) 15-mers primers. PCR primers for AKAP-4 were as follows: forward 5'- GCGTACTCTGATACTACAATGATG -3' and reverse 5'- GGG GTTTTGGGTAAAGTCA- 3' [78]. PCR was performed by 35 amplification cycles at 59°C annealing temperature. For each sample, RNA integrity was checked by amplification of the β-actin cDNA. Successful removal of genomic DNA contamination was confirmed by amplification of the RNA without prior reverse-transcription reaction. All results were confirmed in four independent RT-PCR tests. Immunoblots for AKAP-4 were performed using standard methods, as previously described [78]. Positive controls for immunoblots were proteins extracted from injected MM cells. For healthy mice, protein extracts from human testis were used as positive controls (Applied Biosystems, Foster City, CA, USA) [37].

### Statistical analysis

All data are expressed as mean values ± SEM (Standard Error of the Mean). Results were analyzed using GraphPad Prism software (GraphPad Software, Inc., CA, USA.). Statistical analyses were performed by the two-way ANOVA test. A *p *value < 0.05 was considered statistically significant.

## List of abbreviations used

MM: multiple myeloma; AKAP-4: A kinase anchor protein; RT-PCR: reverse-transcription polymerase chain reaction; ELISA: enzyme-linked immunosorbent assay; Ig: immunoglobulin; NOD/SCID: nonobese diabetes/severe combined immunodeficiency; NRG: NOD-Rag1^null ^IL2rg^null^; NOG: NOD/SCID/γc^null^; SCID-hu: humanized SCID; CTA: cancer/testis antigen; BM: bone marrow; BL: blood; SP: spleen; RT: room temperature.

## Competing interests

The authors declare that they have no competing interests.

## Authors' contributions

LM performed flow-cytometry analyses. YY performed ELISA, RT-PCR experiments, and established the MM model. MJ and CJ participated in study design and coordination, and revised the manuscript. RC analyzed the data and revised the manuscript. EC participated in study design and coordination, and revised the manuscript. MCI and LM carried out the study design, analyzed the data, wrote, and revised the manuscript.   

All authors have read and approved the final manuscript.

## Pre-publication history

The pre-publication history for this paper can be accessed here:

http://www.biomedcentral.com/1471-2407/11/394/prepub

## References

[B1] Altekruse SF Kosary CL Krapcho M Neyman N Aminou R Waldron W Ruhl J Howlader N Tatalovich Z Cho H Mariotto A Eisner MP Lewis DR Cronin K Chen HS Feuer EJ Stinchcomb DG Edwards BK eds SEER Cancer Statistics Review, 1975-2007, National Cancer Institute Bethesda MD

[B2] Kyle RA Gertz MA Witzig TE Lust JA Lacy MQ Dispenzieri A Fonseca R Rajkumar SV Offord JR Larson DR Plevak ME Therneau TM Greipp PR Review of 1027 patients with newly diagnosed multiple myeloma Mayo Clin Proc 2003 78 1 21 33 10.4065/78.1.21 12528874

[B3] Lonial S Cavenagh J Emerging combination treatment strategies containing novel agents in newly diagnosed multiple myeloma Br J Haematol 2009 145 6 681 708 10.1111/j.1365-2141.2009.07649.x 19344388

[B4] Kastritis E Zervas K Symeonidis A Terpos E Delimbassi S Anagnostopoulos N Michali E Zomas A Katodritou E Gika D Pouli A Christoulas D Roussou M Kartasis Z Economopoulos T Dimopoulos MA Improved survival of patients with multiple myeloma after the introduction of novel agents and the applicability of the International Staging System (ISS): an analysis of the Greek Myeloma Study Group (GMSG) Leukemia 2009 23 6 1152 1157 10.1038/leu.2008.402 19225533

[B5] Kumar SK Rajkumar SV Dispenzieri A Lacy MQ Hayman SR Buadi FK Zeldenrust SR Dingli D Russell SJ Lust JA Greipp PR Kyle RA Gertz MA Improved survival in multiple myeloma and the impact of novel therapies Blood 2008 111 5 2516 2520 10.1182/blood-2007-10-116129 17975015 PMC2254544

[B6] Harousseau JL Ten years of improvement in the management of multiple myeloma: 2000-2010 Clin Lymphoma Myeloma Leuk 2010 10 6 424 442 10.3816/CLML.2010.n.076 21156460

[B7] Harousseau JL Attal M Avet-Loiseau H The role of complete response in multiple myeloma Blood 2009 114 15 3139 3146 10.1182/blood-2009-03-201053 19638622

[B8] Bankert RB Hess SD Egilmez NK SCID mouse models to study human cancer pathogenesis and approaches to therapy: potential, limitations, and future directions Front Biosci 2002 1 7 c44 62 10.2741/A758 11915860

[B9] Dalton W Anderson KC Synopsis of a roundtable on validating novel therapeutics for multiple myeloma Clin Cancer Res 2006 12 22 6603 6610 10.1158/1078-0432.CCR-06-1489 17121878

[B10] Epstein J Yaccoby S The SCID-hu myeloma model Methods Mol Med 2005 113 183 190 15968103 10.1385/1-59259-916-8:183

[B11] Mitsiades CS Anderson KC Carrasco DR Mouse models of human myeloma Hematol Oncol Clin North Am 2007 21 6 1051 1069 10.1016/j.hoc.2007.08.003 17996588

[B12] Podar K Tai YT Hideshima T Vallet S Richardson PG Anderson KC Emerging therapies for multiple myeloma Expert Opin Emerg Drugs 2009 14 1 99 127 10.1517/14728210802676278 19249983 PMC3183751

[B13] Cocco C Giuliani N Di Carlo E Ognio E Storti P Abeltino M Sorrentino C Ponzoni M Ribatti D Airoldi I Interleukin-27 acts as multifunctional antitumor agent in multiple myeloma Clin Cancer Res 2010 16 16 4188 4197 10.1158/1078-0432.CCR-10-0173 20587591

[B14] Dai Y Chen S Shah R Pei XY Wang L Almenara JA Kramer LB Dent P Grant S Disruption of Src function potentiates Chk1-inhibitor-induced apoptosis in human multiple myeloma cells in vitro and in vivo Blood 2011 117 6 1947 1957 10.1182/blood-2010-06-291146 21148814 PMC3056642

[B15] Manohar SM Rathos MJ Sonawane V Rao SV Joshi KS Cyclin-dependent kinase inhibitor, P276-00 induces apoptosis in multiple myeloma cells by inhibition of Cdk9-T1 and RNA polymerase II-dependent transcription Leuk Res 2011 7 7 10.1016/j.leukres.2010.12.010 21216463

[B16] de Brito LR Batey MA Zhao Y Squires MS Maitland H Leung HY Hall AG Jackson G Newell DR Irving JA Comparative pre-clinical evaluation of receptor tyrosine kinase inhibitors for the treatment of multiple myeloma Leuk Res 2011 10 10 10.1016/j.leukres.2011.01.011 21316102

[B17] Li J Favata M Kelley JA Caulder E Thomas B Wen X Sparks RB Arvanitis A Rogers JD Combs AP Vaddi K Solomon KA Scherle PA Newton R Fridman JS INCB16562, a JAK1/2 selective inhibitor, is efficacious against multiple myeloma cells and reverses the protective effects of cytokine and stromal cell support Neoplasia 2010 12 1 28 38 20072651 10.1593/neo.91192 PMC2805881

[B18] Tong AW Huang YW Zhang BQ Netto G Vitetta ES Stone MJ Heterotransplantation of human multiple myeloma cell lines in severe combined immunodeficiency (SCID) mice Anticancer Res 1993 13 3 593 597 8391243

[B19] LeBlanc R Catley LP Hideshima T Lentzsch S Mitsiades CS Mitsiades N Neuberg D Goloubeva O Pien CS Adams J Gupta D Richardson PG Munshi NC Anderson KC Proteasome inhibitor PS-341 inhibits human myeloma cell growth in vivo and prolongs survival in a murine model Cancer Res 2002 62 17 4996 5000 12208752

[B20] Podar K Tonon G Sattler M Tai YT Legouill S Yasui H Ishitsuka K Kumar S Kumar R Pandite LN Hideshima T Chauhan D Anderson KC The small-molecule VEGF receptor inhibitor pazopanib (GW786034B) targets both tumor and endothelial cells in multiple myeloma Proc Natl Acad Sci USA 2006 103 51 19478 19483 10.1073/pnas.0609329103 17164332 PMC1748251

[B21] Navas TA Nguyen AN Hideshima T Reddy M Ma JY Haghnazari E Henson M Stebbins EG Kerr I O'Young G Kapoun AM Chakravarty S Mavunkel B Perumattam J Luedtke G Dugar S Medicherla S Protter AA Schreiner GF Anderson KC Higgins LS Inhibition of p38alpha MAPK enhances proteasome inhibitor-induced apoptosis of myeloma cells by modulating Hsp27, Bcl-X(L), Mcl-1 and p53 levels in vitro and inhibits tumor growth in vivo Leukemia 2006 20 6 1017 1027 10.1038/sj.leu.2404200 16617327

[B22] Carlo-Stella C Guidetti A Di Nicola M Longoni P Cleris L Lavazza C Milanesi M Milani R Carrabba M Farina L Formelli F Gianni AM Corradini P CD52 antigen expressed by malignant plasma cells can be targeted by alemtuzumab in vivo in NOD/SCID mice Exp Hematol 2006 34 6 721 727 10.1016/j.exphem.2006.03.005 16728276

[B23] Baughn LB Di Liberto M Wu K Toogood PL Louie T Gottschalk R Niesvizky R Cho H Ely S Moore MA Chen-Kiang S A novel orally active small molecule potently induces G1 arrest in primary myeloma cells and prevents tumor growth by specific inhibition of cyclin-dependent kinase 4/6 Cancer Res 2006 66 15 7661 7667 10.1158/0008-5472.CAN-06-1098 16885367

[B24] Watanabe M Dewan MZ Okamura T Sasaki M Itoh K Higashihara M Mizoguchi H Honda M Sata T Watanabe T Yamamoto N Umezawa K Horie R A novel NF-kappaB inhibitor DHMEQ selectively targets constitutive NF-kappaB activity and induces apoptosis of multiple myeloma cells in vitro and in vivo Int J Cancer 2005 114 1 32 38 10.1002/ijc.20688 15523684

[B25] Dewan MZ Watanabe M Terashima K Aoki M Sata T Honda M Ito M Yamaoka S Watanabe T Horie R Yamamoto N Prompt tumor formation and maintenance of constitutive NF-kappaB activity of multiple myeloma cells in NOD/SCID/gammacnull mice Cancer Sci 2004 95 7 564 568 10.1111/j.1349-7006.2004.tb02487.x 15245591 PMC11159879

[B26] Pearson T Shultz LD Miller D King M Laning J Fodor W Cuthbert A Burzenski L Gott B Lyons B Foreman O Rossini AA Greiner DL Non-obese diabetic-recombination activating gene-1 (NOD-Rag1 null) interleukin (IL)-2 receptor common gamma chain (IL2r gamma null) null mice: a radioresistant model for human lymphohaematopoietic engraftment Clin Exp Immunol 2008 154 2 270 284 10.1111/j.1365-2249.2008.03753.x 18785974 PMC2612717

[B27] Mitsiades CS Mitsiades NS Bronson RT Chauhan D Munshi N Treon SP Maxwell CA Pilarski L Hideshima T Hoffman RM Anderson KC Fluorescence imaging of multiple myeloma cells in a clinically relevant SCID/NOD in vivo model: biologic and clinical implications Cancer Res 2003 63 20 6689 6696 14583463

[B28] Asosingh K De Raeve H Van Riet I Van Camp B Vanderkerken K Multiple myeloma tumor progression in the 5T2MM murine model is a multistage and dynamic process of differentiation, proliferation, invasion, and apoptosis Blood 2003 101 8 3136 3141 10.1182/blood-2002-10-3000 12480692

[B29] Chantry AD Heath D Mulivor AW Pearsall S Baud'huin M Coulton L Evans H Abdul N Werner ED Bouxsein ML Key ML Seehra J Arnett TR Vanderkerken K Croucher P Inhibiting activin-A signaling stimulates bone formation and prevents cancer-induced bone destruction in vivo Journal of Bone and Mineral Research 2010 25 12 2633 2646 10.1002/jbmr.142 20533325

[B30] Campbell RA Sanchez E Steinberg J Shalitin D Li ZW Chen H Berenson JR Vorinostat enhances the antimyeloma effects of melphalan and bortezomib Eur J Haematol 2010 84 3 201 211 10.1111/j.1600-0609.2009.01384.x 19929977

[B31] Sordillo EM Pearse RN RANK-Fc: a therapeutic antagonist for RANK-L in myeloma Cancer 2003 97 3 Suppl 802 812 12548579 10.1002/cncr.11134

[B32] Campbell RA Sanchez E Steinberg JA Baritaki S Gordon M Wang C Shalitin D Chen H Pang S Bonavida B Said J Berenson JR Antimyeloma effects of arsenic trioxide are enhanced by melphalan, bortezomib and ascorbic acid Br J Haematol 2007 138 4 467 478 10.1111/j.1365-2141.2007.06675.x 17587338

[B33] Turner RM Johnson LR Haig-Ladewig L Gerton GL Moss SB An X-linked gene encodes a major human sperm fibrous sheath protein, hAKAP82. Genomic organization, protein kinase A-RII binding, and distribution of the precursor in the sperm tail J Biol Chem 1998 273 48 32135 32141 10.1074/jbc.273.48.32135 9822690

[B34] Turner RM Musse MP Mandal A Klotz K Jayes FC Herr JC Gerton GL Moss SB Chemes HE Molecular genetic analysis of two human sperm fibrous sheath proteins, AKAP4 and AKAP3, in men with dysplasia of the fibrous sheath J Androl 2001 22 2 302 315 11229805

[B35] Chiriva-Internati M Cobos E Da Silva DM Kast WM Sperm fibrous sheath proteins: a potential new class of target antigens for use in human therapeutic cancer vaccines Cancer Immun 2008 8 8 18433090 PMC2935778

[B36] Scanlan MJ Gure AO Jungbluth AA Old LJ Chen YT Cancer/testis antigens: an expanding family of targets for cancer immunotherapy Immunol Rev 2002 188 22 32 10.1034/j.1600-065X.2002.18803.x 12445278

[B37] Chiriva-Internati M Ferrari R Yu Y Hamrick C Gagliano N Grizzi F Frezza E Jenkins MR Hardwick F D'Cunha N Kast WM Cobos E AKAP-4: a novel cancer testis antigen for multiple myeloma Br J Haematol 2008 140 4 465 8 10.1111/j.1365-2141.2007.06940.x 18217892

[B38] Miyakawa Y Ohnishi Y Tomisawa M Monnai M Kohmura K Ueyama Y Ito M Ikeda Y Kizaki M Nakamura M Establishment of a new model of human multiple myeloma using NOD/SCID/gammac(null) (NOG) mice Biochem Biophys Res Commun 2004 313 2 258 262 10.1016/j.bbrc.2003.11.120 14684154

[B39] Yaccoby S Barlogie B Epstein J Primary myeloma cells growing in SCID-hu mice: a model for studying the biology and treatment of myeloma and its manifestations Blood 1998 92 8 2908 2913 9763577

[B40] Dutta-Simmons J Zhang Y Gorgun G Gatt M Mani M Hideshima T Takada K Carlson NE Carrasco DE Tai YT Raje N Letai AG Anderson KC Carrasco DR Aurora kinase A is a target of Wnt/beta-catenin involved in multiple myeloma disease progression Blood 2009 114 13 2699 2708 19652203 10.1182/blood-2008-12-194290

[B41] Azab AK Runnels JM Pitsillides C Moreau AS Azab F Leleu X Jia X Wright R Ospina B Carlson AL Alt C Burwick N Roccaro AM Ngo HT Farag M Melhem MR Sacco A Munshi NC Hideshima T Rollins BJ Anderson KC Kung AL Lin CP Ghobrial IM CXCR4 inhibitor AMD3100 disrupts the interaction of multiple myeloma cells with the bone marrow microenvironment and enhances their sensitivity to therapy Blood 2009 113 18 4341 4351 10.1182/blood-2008-10-186668 19139079 PMC2676090

[B42] Labrinidis A Diamond P Martin S Hay S Liapis V Zinonos I Sims NA Atkins GJ Vincent C Ponomarev V Findlay DM Zannettino AC Evdokiou A Apo2L/TRAIL inhibits tumor growth and bone destruction in a murine model of multiple myeloma Clin Cancer Res 2009 15 6 1998 2009 10.1158/1078-0432.CCR-08-2444 19276263 PMC5573683

[B43] Reijmers RM Groen RW Rozemuller H Kuil A de Haan-Kramer A Csikos T Martens AC Spaargaren M Pals ST Targeting EXT1 reveals a crucial role for heparan sulfate in the growth of multiple myeloma Blood 2010 115 3 601 604 10.1182/blood-2009-02-204396 19965677

[B44] Nakashima T Ishii T Tagaya H Seike T Nakagawa H Kanda Y Akinaga S Soga S Shiotsu Y New molecular and biological mechanism of antitumor activities of KW-2478, a novel nonansamycin heat shock protein 90 inhibitor, in multiple myeloma cells Clin Cancer Res 2010 16 10 2792 2802 10.1158/1078-0432.CCR-09-3112 20406843

[B45] Lavazza C Carlo-Stella C Giacomini A Cleris L Righi M Sia D Di Nicola M Magni M Longoni P Milanesi M Francolini M Gloghini A Carbone A Formelli F Gianni AM Human CD34+ cells engineered to express membrane-bound tumor necrosis factor-related apoptosis-inducing ligand target both tumor cells and tumor vasculature Blood 2010 115 11 2231 2240 10.1182/blood-2009-08-239632 20075160

[B46] Koomen JM Haura EB Bepler G Sutphen R Remily-Wood ER Benson K Hussein M Hazlehurst LA Yeatman TJ Hildreth LT Sellers TA Jacobsen PB Fenstermacher DA Dalton WS Proteomic contributions to personalized cancer care Mol Cell Proteomics 2008 7 10 1780 1794 10.1074/mcp.R800002-MCP200 18664563 PMC2559938

[B47] Yeung J Chang H Genomic aberrations and immunohistochemical markers as prognostic indicators in multiple myeloma J Clin Pathol 2008 61 7 832 836 10.1136/jcp.2007.049585 18077770

[B48] Croese JW Vas Nunes CM Radl J van den Enden-Vieveen MH Brondijk RJ Boersma WJ The 5T2 mouse multiple myeloma model: characterization of 5T2 cells within the bone marrow Br J Cancer 1987 56 5 555 560 10.1038/bjc.1987.241 3426918 PMC2001900

[B49] Vanderkerken K De Raeve H Goes E Van Meirvenne S Radl J Van Riet I Thielemans K Van Camp B Organ involvement and phenotypic adhesion profile of 5T2 and 5T33 myeloma cells in the C57BL/KaLwRij mouse Br J Cancer 1997 76 4 451 460 10.1038/bjc.1997.409 9275021 PMC2227997

[B50] Campbell RA Manyak SJ Yang HH Sjak-Shie NN Chen H Gui D Popoviciu L Wang C Gordon M Pang S Bonavida B Said J Berenson JR LAGlambda-1: a clinically relevant drug resistant human multiple myeloma tumor murine model that enables rapid evaluation of treatments for multiple myeloma Int J Oncol 2006 28 6 1409 1417 16685443

[B51] Kovalchuk AL Kim JS Park SS Coleman AE Ward JM Morse HC Kishimoto T Potter M Janz S IL-6 transgenic mouse model for extraosseous plasmacytoma Proc Natl Acad Sci USA 2002 99 3 1509 1514 10.1073/pnas.022643999 11805288 PMC122221

[B52] Cheung WC Kim JS Linden M Peng L Van Ness B Polakiewicz RD Janz S Novel targeted deregulation of c-Myc cooperates with Bcl-X(L) to cause plasma cell neoplasms in mice J Clin Invest 2004 113 12 1763 1773 15199411 10.1172/JCI20369 PMC420503

[B53] Yaccoby S Epstein J The proliferative potential of myeloma plasma cells manifest in the SCID-hu host Blood 1999 94 10 3576 3582 10552969

[B54] Yaccoby S Johnson CL Mahaffey SC Wezeman MJ Barlogie B Epstein J Antimyeloma efficacy of thalidomide in the SCID-hu model Blood 2002 100 12 4162 4168 10.1182/blood-2002-03-0939 12393672

[B55] Pearse RN Sordillo EM Yaccoby S Wong BR Liau DF Colman N Michaeli J Epstein J Choi Y Multiple myeloma disrupts the TRANCE/osteoprotegerin cytokine axis to trigger bone destruction and promote tumor progression Proc Natl Acad Sci USA 2001 98 20 11581 11586 10.1073/pnas.201394498 11562486 PMC58772

[B56] Araki K Sangai T Miyamoto S Maeda H Zhang SC Nakamura M Ishii G Hasebe T Kusaka H Akiyama T Tokuda Y Nagai K Minami H Ochiai A Inhibition of bone-derived insulin-like growth factors by a ligand-specific antibody suppresses the growth of human multiple myeloma in the human adult bone explanted in NOD/SCID mouse Int J Cancer 2006 118 10 2602 2608 10.1002/ijc.21653 16353147

[B57] Bueno C Lopes LF Greaves M Menendez P Toward development of a novel NOD/SCID-based in vivo strategy to model multiple myeloma pathogenesis Exp Hematol 2007 35 10 1477 8 Epub 2007 Aug 3 10.1016/j.exphem.2007.06.012 17681665

[B58] van den Akker TW Radl J Franken-Postma E Hagemeijer A Cytogenetic findings in mouse multiple myeloma and Waldenstrom's macroglobulinemia Cancer Genet Cytogenet 1996 86 2 156 161 10.1016/0165-4608(95)00169-7 8603345

[B59] Radl J Punt YA van den Enden-Vieveen MH Bentvelzen PA Bakkus MH van den Akker TW Benner R The 5T mouse multiple myeloma model: absence of c-myc oncogene rearrangement in early transplant generations Br J Cancer 1990 61 2 276 278 10.1038/bjc.1990.51 2310679 PMC1971422

[B60] Fernandes MS Gomes EM Butcher LD Hernandez-Alcoceba R Chang D Kansopon J Newman J Stone MJ Tong AW Growth inhibition of human multiple myeloma cells by an oncolytic adenovirus carrying the CD40 ligand transgene Clin Cancer Res 2009 15 15 4847 4856 10.1158/1078-0432.CCR-09-0451 19622582

[B61] Chauhan D Singh AV Aujay M Kirk CJ Bandi M Ciccarelli B Raje N Richardson P Anderson KC A novel orally active proteasome inhibitor ONX 0912 triggers in vitro and in vivo cytotoxicity in multiple myeloma Blood 2010 116 23 4906 4915 10.1182/blood-2010-04-276626 20805366 PMC3321748

[B62] Singh AV Bandi M Aujay MA Kirk CJ Hark DE Raje N Chauhan D Anderson KC PR-924, a selective inhibitor of the immunoproteasome subunit LMP-7, blocks multiple myeloma cell growth both in vitro and in vivo Br J Haematol 2011 152 2 155 163 10.1111/j.1365-2141.2010.08491.x 21114484 PMC3138210

[B63] Nefedova Y Landowski TH Dalton WS Bone marrow stromal-derived soluble factors and direct cell contact contribute to de novo drug resistance of myeloma cells by distinct mechanisms Leukemia 2003 17 6 1175 1182 10.1038/sj.leu.2402924 12764386

[B64] Urashima M Chen BP Chen S Pinkus GS Bronson RT Dedera DA Hoshi Y Teoh G Ogata A Treon SP Chauhan D Anderson KC The development of a model for the homing of multiple myeloma cells to human bone marrow Blood 1997 90 2 754 765 9226176

[B65] Barlogie B Shaughnessy J Tricot G Jacobson J Zangari M Anaissie E Walker R Crowley J Treatment of multiple myeloma Blood 2004 103 1 20 32 10.1182/blood-2003-04-1045 12969978

[B66] Hideshima T Bergsagel PL Kuehl WM Anderson KC Advances in biology of multiple myeloma: clinical applications Blood 2004 104 3 607 618 10.1182/blood-2004-01-0037 15090448

[B67] Steensma DP Gertz MA Greipp PR Kyle RA Lacy MQ Lust JA Offord JR Plevak MF Therneau TM Witzig TE A high bone marrow plasma cell labeling index in stable plateau-phase multiple myeloma is a marker for early disease progression and death Blood 2001 97 8 2522 2523 10.1182/blood.V97.8.2522 11290618

[B68] Vande Broek I Vanderkerken K Van Camp B Van Riet I Extravasation and homing mechanisms in multiple myeloma Clin Exp Metastasis 2008 25 4 325 334 10.1007/s10585-007-9108-4 17952614

[B69] Moulopoulos LA Dimopoulos MA Vourtsi A Gouliamos A Vlahos L Bone lesions with soft-tissue mass: magnetic resonance imaging diagnosis of lymphomatous involvement of the bone marrow versus multiple myeloma and bone metastases Leuk Lymphoma 1999 34 1-2 179 184 10350347 10.3109/10428199909083395

[B70] Santonocito AM Consoli U Bagnato S Milone G Palumbo GA Di Raimondo F Stagno F Guglielmo P Giustolisi R Flow cytometric detection of aneuploid CD38(++) plasmacells and CD19(+) B-lymphocytes in bone marrow, peripheral blood and PBSC harvest in multiple myeloma patients Leuk Res 2004 28 5 469 477 10.1016/j.leukres.2003.09.015 15068900

[B71] Tong WG Chen R Plunkett W Siegel D Sinha R Harvey RD Badros AZ Popplewell L Coutre S Fox JA Mahadocon K Chen T Kegley P Hoch U Wierda WG Phase I and pharmacologic study of SNS-032, a potent and selective Cdk2, 7, and 9 inhibitor, in patients with advanced chronic lymphocytic leukemia and multiple myeloma J Clin Oncol 2010 28 18 3015 3022 10.1200/JCO.2009.26.1347 20479412 PMC4979218

[B72] Hall MN Jagannathan JP Ramaiya NH Shinagare AB Van den Abbeele AD Imaging of extraosseous myeloma: CT, PET/CT, and MRI features AJR Am J Roentgenol 2010 195 5 1057 1065 10.2214/AJR.10.4384 20966307

[B73] Winterbottom AP Shaw AS Imaging patients with myeloma Clin Radiol 2009 64 1 1 11 10.1016/j.crad.2008.07.006 19070692

[B74] Rabin N Kyriakou C Coulton L Gallagher OM Buckle C Benjamin R Singh N Glassford J Otsuki T Nathwani AC Croucher PI Yong KL A new xenograft model of myeloma bone disease demonstrating the efficacy of human mesenchymal stem cells expressing osteoprotegerin by lentiviral gene transfer Leukemia 2007 21 10 2181 2191 10.1038/sj.leu.2404814 17657224

[B75] Gatt ME Zhao JJ Ebert MS Zhang Y Chu Z Mani M Gazit R Carrasco DE Dutta-Simmons J Adamia S Minvielle S Tai YT Munshi NC Avet-Loiseau H Anderson KC Carrasco DR MicroRNAs 15a/16-1 function as tumor suppressor genes in multiple myeloma Blood 2010 10.1182/blood-2009-11-253294 20962322

[B76] de Weers M Tai YT van der Veer MS Bakker JM Vink T Jacobs DC Oomen LA Peipp M Valerius T Slootstra JW Mutis T Bleeker WK Anderson KC Lokhorst HM van de Winkel JG Parren PW Daratumumab, a novel therapeutic human CD38 monoclonal antibody, induces killing of multiple myeloma and other hematological tumors J Immunol 2011 186 3 1840 1848 10.4049/jimmunol.1003032 21187443

[B77] Lim SH Wang Z Chiriva-Internati M Xue Y Sperm protein 17 is a novel cancer-testis antigen in multiple myeloma Blood 2001 97 5 1508 1510 10.1182/blood.V97.5.1508 11222401

[B78] Towbin H Staehelin T Gordon J Electrophoretic transfer of proteins from polyacrylamide gels to nitrocellulose sheets: procedure and some applications Proc Natl Acad Sci USA 1979 76 9 4350 4354 10.1073/pnas.76.9.4350 388439 PMC411572

